# Investigation and Comparison of the Effect of TGF-β3, kartogenin and Avocado/Soybean Unsaponifiables on the In-vitro and In-vivo Chondrogenesis of Human Adipose-Derived Stem Cells on Fibrin Scaffold

**DOI:** 10.22037/ijpr.2020.114420.14851

**Published:** 2021

**Authors:** Batool Hashemibeni, Mohammad Ali Izadi, Ali Valiani, Ibrahim Esfandiari, Hamid Bahramian, Hengameh Dortaj, Majid Pourentezari

**Affiliations:** a *Department of Anatomical Sciences and Molecular Biology, Faculty of Medicine, Isfahan University of Medical Sciences, Isfahan, Iran. *; b *Department of Tissue Engineering and Applied Cell Science, Faculty of Applied Medical Science and Technologies, Shiraz University, Shiraz, Iran. *; c *Department of Biology and Anatomical Sciences, Faculty of Medicine, Shahid Sadoughi University of Medical Sciences, Yazd, Iran.*

**Keywords:** TGFβ3, Avocado/Soybean, Kartogenin, Human adipose-derived stem cells, Fibrin, Chondrogenesis

## Abstract

Due to the lack of suitable therapeutic approaches to cartilage defect, the objective of this study was to determine the effect of Transforming growth factor-β3 (TGF-β3), avocado/soybean (ASU) and Kartogenin (KGN) on chondrogenic differentiation in human adipose-derived stem cells (hADSCs) on fibrin scaffold. hADSCs seeded in fibrin scaffold and cultured in chondrogenic media. These cells were divided into 4 groups (control, TGF-β3, ASU and KGN). Cell viability was estimated by MTT assay. Differentiated cells were evaluated by histological and immunohistochemical (IHC) techniques. Expression genes [*sex determining region Y-box 9* (*SOX9*), *Aggrecan* (*AGG*), *type II collagen* (*Coll II*) and *type X collagen* (*Coll X*)] were assessed by real-time PCR. For a study on an animal model, differentiated cells in fibrin scaffolds were subcutaneously transplanted in rats. Histological and immunohistochemistry were done in the animal model. The results of the real-time PCR indicated that *SOX9*, *AGG* and *Col II* genes expression in TGF-β3, KGN and ASU groups were significantly higher (*p* < 0.01) compared to the control group, *Col X* gene expression only in the TGF-β3 group was significantly higher (*p* *< *0.01) compared to the control group. The glycosaminoglycan (GAG) deposition was higher in TGF-β3, KGN and ASU groups compared to the control group. The immunohistological analysis showed the distribution of collagen type X in the extracellular matrix in the fibrin scaffold TGF-β3 group was significantly higher in control, KGN and ASU groups, and (*p* < 0.001). ASU, particularly KGN, was suitable for successful chondrogenic differentiation of hADSCs and a suppressor of the consequent hypertrophy.

## Introduction

Articular cartilage is an avascular tissue. This property, combined with the low proliferation and motility of the chondrocytes, means that it has a low capacity to regenerate when it degrades or is damaged through injury (1). Osteoarthritis (OA) is the most common and disabling rheumatic disease, affecting millions of people worldwide (2). It is the major cause of joint pain, results in substantial morbidity and disability, and imposes a great economic burden on society (2). Despite the high prevalence and societal burden of OA, there is no cure for it yet. 

A recent tissue engineering approach to develop such an implant has exploited additive manufacturing techniques to seed cells within a hydrogels construct reinforced with a three-dimensional (3D)-printed lattice of polymer fibers. The seeded cell population consists of mesenchymal stem cells (MSCs) and/or chondrocytes which are then biochemically and/or mechanically stimulated to promote chondrogenic differentiation of the MSCs and enhance matrix deposition by the chondrocytes (3).

TGF-β plays an important role in both natural cartilages and in approaches to engineer artificial cartilage. It is produced in-vivo by chondrocytes and binds rapidly to the extracellular matrix (ECM) for storage. After undergoing an activation process, an active form of TGF-β is released from this stored state and can freely diffuse. It then acts to stimulate chondrocytes to synthesis ECM components, including collagen type II (Coll II) and proteoglycans. Importantly for tissue engineering applications, it also drives MSCs to differentiate into chondrocytes (1). But this agent has adverse effects on cells as well as they are expensive, and they have a short half time (1, 4).

Natural products can be safer than prescription medications with fewer undesirable side effects. For this reason, there is great interest in the application of herbal agents for the treatment of diseases. Avocado/Soybean Unsaponifiables (ASU) is natural vegetable extracts made from avocado and soybean oils, consisting of the leftover fraction (approximately 1%) that cannot be made into soap after saponification. ASU is composed of one-third avocado and two-thirds soybean Unsaponifiables. The major components of ASU are phytosterols β-sitosterol, campesterol, and stigmasterol, which are rapidly incorporated into cells. ASU is a complex mixture of many compounds, including fat-soluble vitamins, sterols, triterpene alcohols, and possibly furan fatty acids. The identity of the active component(s) remains unknown. The sterol contents of ASU preparations are the primary contributors to biological activity in articular chondrocytes (5). Preclinical *in-vitro* and *in-vivo* studies have demonstrated that ASUs have beneficial effects on OA (6, 7).

Kartogenin (KGN) is a small heterocyclic molecule that enhances chondrocytes differentiation of primary MSCs through up-regulation of chondrogenic gene expression and characteristic chondrocytes activities (8). Recently, the small molecule KGN was reported to promote the differentiation of bone marrow MSCs into chondrocytes in culture (9). KGN also showed chondroprotective effects when injected intraarticularly in two mouse models of OA (10).

Different stem cell sources are frequently used in tissue engineering, including bone marrow stem cells (BM-MSCs) and adipose-derived stem cells (ADSCs). BM-MSCs were considered the main cellular source of tissue engineering for a good long time, but recently, ADSCs have received extensive attention due to their availability, less invasive nature and high chondrogenic potential (7).

Biological materials have a crucial role in tissue engineering. In this regard, various naturally derived and synthetic scaffolds have been used for tissue repair (11). A rewarding scaffold should be biodegradable, biocompatible, and porous. Besides, it must provide suitable conditions for the adhesion, proliferation, and migration of chondrogenic cells (7, 12). A fibrin scaffold is a network of proteins that holds a variety of living tissues together. Fibrin has unique biocompatibility and viscoelasticity properties, but its sustainability is weak and degrades rapidly (13). Fibrin scaffold facilitates cell proliferation and migration, the transfer of molecules and food, as well as the disposal of metabolites (14).

Due to some of the major unsolved challenges in the chondrogenesis process are Transforming growth factor-βs (TGF-βs) disadvantageous effects. The purpose of the present study was to compare the effectiveness of TGF-β3, ASU and KGN on chondrogenic differentiation in hADSCs on fibrin scaffold *in-vitro *and animal models.

## Experimental

*Isolation and culture of human ADSCs*

The samples of subcutaneous abdominal adipose tissue were harvested from three people aged 25–40 years who underwent scheduled liposuction. The study was approved by the Institutional Ethics Committee of Isfahan University of Medical Sciences (IR:MUI.REC.1395.3.176). The adipose tissue was digested by collagenase type IA solution at 37 °C for 30 min. Subsequent to that, a complete cell culture medium [Dulbecco’s Modified Eagle Medium (DMEM) supplemented with 10% fetal bovine serum (FBS) and 1% penicillin/streptomycin (Gibco, America)) was added to the cell suspension to neutralize the activity of the enzyme. Then, the cell suspension was centrifuged at 1400 rpm for 7 min, and the supernatant was removed along with adipocytes. Finally, the resulting cellular pellet was cultured in a complete cell culture medium at 37 °C, 5% CO2 conditions (7). Additional cells were removed by changing the medium after 24 h.

*Preparation of Fibrinogen and Thrombin*

Fresh frozen plasma solutions (FFP) and fibrinogen were obtained from The Isfahan Blood Transfusion Organization. FFP was defrosted in a water bath at 37 °C, and then 15 mL of it was mixed with 10 mL of gluconate calcium. The acquired solution was incubated for 3 h at 37 °C and then centrifuged at 2500 rpm for 10 min. The supernatant was harvested as thrombin. Fibrinogen and thrombin solutions were prepared for use as cell culture.

*In-vitro Chondrogenic Differentiation*

The hADSCs at a concentration of 1 × 10^6^ cells were liquefied within fibrinogen, and thrombin with an equal amount (350 μL) was added to them. At that time, the fibrin scaffolds in chondrogenic medium containing DMEM-high glucose (Gibco, America) along with 1% insulin-transferrin-selenium (Sigma, America), 50 μg/mL ascorbate 2-phosphate, dexamethasone 100 nmol (Sigma, America), 50 mg/mL bovine serum albumin (BSA) (Sigma, America), 0.5 mL linoleic acid (Sigma, America), 1% penicillin/streptomycin (Sigma, America) without any growth factor as the control group and chondrogenic medium added 10 μg/mL ASU (Perarin Pars, Iran) (15), 100 nmol KGN (Sigma, America) (16) and 10 ng/mL TGF-β3(Sigma, America) (17) as the treatment groups were located in the incubator (37 °C, 5% CO2, 99% humidity) for 14 days. Then 500 μL of the chondrogenic medium was added to each well. The half amount of medium was substituted every 3 days (11).

*Cell Viability Assay*

The viability of hADSCs in the fibrin scaffold among different groups was assessed by the 3-(4, 5-dimethylthiazol-2-yl)-2, 5-diphenyltetrazolium bromide (MTT) assay on day 14. First, the medium of each well was removed, washed with PBS, and replaced with 400 μL serum-free medium and 40 μL MTT solutions (5 mg/mL in PBS). Then, it was incubated for 4 h at 37 °C, 5% CO_2_. The medium was then discarded, 400 μL Dimethyl sulfoxide (DMSO) (Sigma, America) was added to each well, and the scaffold was incubated in the dark for 2 h. A purple color was created by DMSO after it dissolved the formazan crystals. Then 100 μL of the solution was transferred to a 96-well plate, and the absorbance of each well was read at 570 nm with an ELISA reader (Hiperion MPR4). MTT assay was also applied to the scaffolds without cells as controls, and then the control values were subtracted from the measured values. The assays were performed in triplicate (18).

*Histological Examination*

On the 14th day, after the chondrogenic induction, samples of hADSCs that were seeded in fibrin scaffolds were collected, fixed with 10% formal saline for 24 h, dehydrated by a graded series of ethyl alcohol, cleared in xylem and embedded in paraffin. Sections were cut at 4 mm and located on microscope slides. Safranin O (Merck, Germany), Toluidine blue (Merck, Germany) and hematoxylin and eosin (H&E; Merck, Germany) were selected for staining. Toluidine blue and Safranin O were used to estimate the ECM components of cartilage glycosaminoglycan (GAG) deposition, and hematoxylin and eosin were used to reveal the other structures. Safranin O staining consists of staining the sections with Wiegert’s iron hematoxylin working for 15 min, fast green (Merck, Germany) for 6 min and 1% safranin O for 6 min. Subsequently, in each staining step, the sections were washed and then rinsed in absolute ethyl alcohol. For Toluidine blue staining, the sections were plunged in for 5 min. For H&E staining, the sections were stained with Harris hematoxylin (Merck, Germany) for 15 min, washed in running water, differentiated in acid alcohol 1%, and then a blue stain enhancement was done by immersion in 0.05% carbonate lithium (Sigma, America) for 5 s. then the sections were washed in running water and stained in Eosin-Y (Merck, Germany) for 3 min. Finally, all slides were dehydrated by immersion in ethyl alcohol and cleared in xylene, then mounting using Microscopy Entellan (Merck, Germany) for later observation under a light microscope (Olympus BX43); the stained sections were seen (19).

*Immunohistochemistry (IHC) Assay*

IHC was done on the samples on the 14th day. The samples were fixed in 10% formal saline for 24 h and then embedded in paraffin and sectioned at 4 μL. Antigen retrieval for collagen II was done by incubation with 8 mg/mL hyaluronidase (Sigma, America) for 3 h at 37 °C, but antigen retrieval for collagen X (Coll X) required 2 mg/mL hyaluronidase (Sigma, America) for 2 h. Furthermore, Coll X samples were treated with 1 mg/mL Pronase (Sigma, America). With blocking buffer, the nonspecific c binding sites were blocked and sections were incubated with primary antibodies at 4 °C overnight. Monoclonal antibodies directed against human antigens were available for Coll II (ab3092; Abcam, America) or Coll X (C7974; Sigma, America). Sections were washed and incubated with the anti-mouse IgG secondary antibody (ab2891; Abcam, America) that was connected to horseradish peroxidase and was established using 3,3′- diaminobenzene (DAB) substrate kit (ab94656; Abcam, America) (20). To semi-quantify the immunoreactivity of Coll II and Coll X in KGN, TGF-β3 and the control groups on day 14, the images from each group were measured using the ImageJ software (version 1.8.0_112).

*RNA Isolation and Real-Time Polymerase Chain Reaction (PCR)*

Real-time quantitative PCR was performed to estimate mRNA expression of *Coll* *I*, *Coll II* and *Coll X*, *AGG*, and *SOX9* genes in hADSCs quantitatively among different groups. Total RNA was isolated by RNeasy mini kit (Qiagen, Germany) and treated with RNase-free DNase set (Qiagen, Germany) to eliminate genomic DNA. RNA concentration was determined using a BioPhotometer (Eppendorf). ACCORDING TO THE MANUFACTURER’S INSTRUCTIONS, total RNA (100 ng) was reverse-transcribed to cDNA by Revert Aid™ First Strand cDNA Synthesis Kit (Fermentas, Germany). Maxima SYBR Green Rox qPCR master mix kit (Fermentas, Germany) was used for real-time Real-time PCR. Primer sequences are shown in Table 1. Real-time PCR reactions were performed using the Comparative Ct (∆∆Ct) method. The relative expression level of the genes was computed by calculating the ratio of the amount of the genes to that of the endogenous control (GAPDH). A melting curve was produced to determine the melting temperature of specific amplification. These experiments were carried out in triplicate. cDNA was amplified under the following conditions: denaturation at 95 °C for 10 min, denaturation at 95 °C for 15 secs, annealing at 60 °C for one min and extension at 72 °C for one min; the whole process was performed for 40 cycles (Table 1) (7).

*Statistical Analysis*

A one-way ANOVA test was used to evaluate the differences between groups, and the Tukey post-hoc test was operated for the determination of differences between every two groups. The term ‘statistically significant’ was used to signify a two-sided *p*-value < 0.05

## Results

*MTT assay*

**Figure 1:**
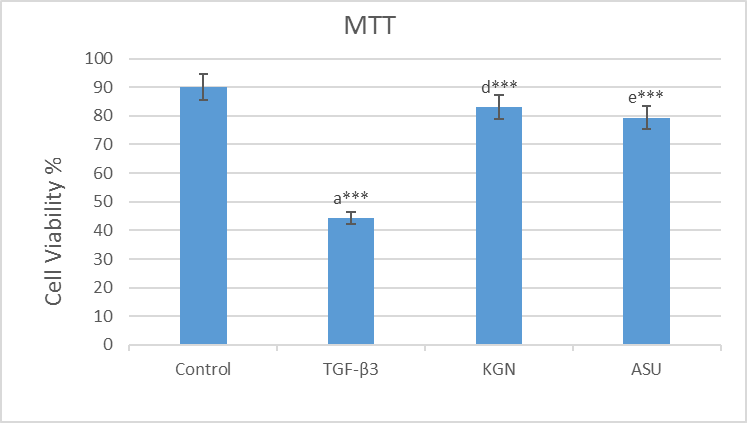
MTT assay results in 14 days after the culture of hADSCs in chondrogenic medium supplemented with TGF-β3, KGN and ASU fibrin scaffold. ^*^P < 0.05, ^**^P < 0.01, ^***^P < 0.001. a: Difference between control and TGF-β3. b: Difference between control and KGN. c: Difference between control and ASU. d: Difference between TGF-β3 and KGN. e: Difference between TGF-β3 and ASU. f: Difference between KGN and ASU

After the use of MTT solution, the blue-black formazan crystals were seen in hADSCs, which demonstrated their metabolic activity. The MTT assay results are exhibited. Applying KGN and ASU decreased the proliferation and viability of hADSCs. However, the evaluation of the results showed that it did not have significant differences compared to the control group (*p *> 0.05), but in the group that was treated by TGF-β3, decreasing of cell viability had a significant difference (*p < *0.001) compared to the control group (Figure 1). 

*Real-Time PCR*

**Figure 2:**
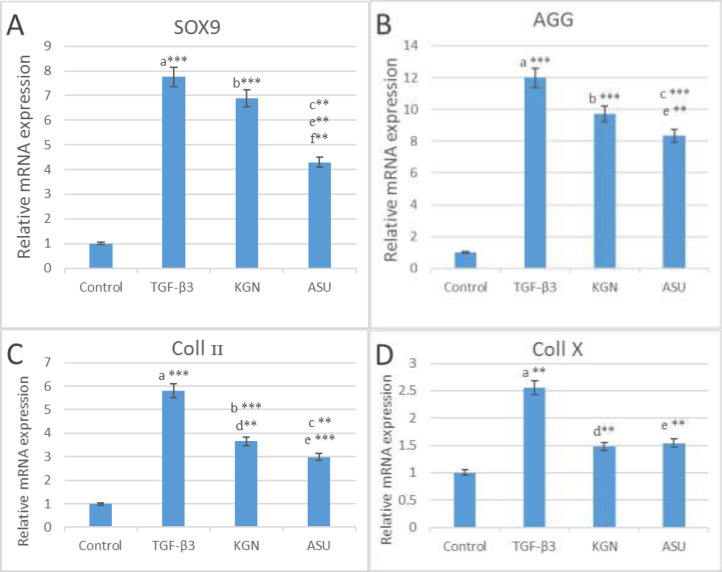
The results of (A)* SOX9*, (B)* AGG*, (C) *Coll II* and (D) *Coll X* genes expression in control, TGF β3, KGN and ASU groups 14 days after the culture of hADSCs. Data are presented as mean ± SD. Error bars represent the standard deviation of the mean. ^*^P < 0.05, ^**^P < 0.01, ^***^P < 0.001. RQ (Relative quantification) indicates the relative level of gene expression. ^a^Difference between control and TGF-β3. ^b^Difference between control and KGN. ^c^Difference between control and ASU. ^d^Difference between TGF-β3 and KGN. ^e^Difference between TGF-β3 and ASU. ^f^Difference between KGN and ASU

The results of the real-time PCR indicated that *SOX9*, *AGG* and *Coll II* genes expression in TGF-β3, KGN and ASU groups were significantly higher (*p < *0.01) than in the control group, but *Coll X* gene expression only in the TGF-β3 group was significantly higher (*P < *0.01) than in the control group. Also, the results of the real-time PCR showed that *SOX9* gene expression in the KGN group is significantly higher (*p < *0.01) than in the ASU group. But that *AGG*, *Coll II* and *Coll X* gene expression did not have significant differences between KGN and ASU groups (*p* > 0.05) (Figure 2).

*Histological examination In-vitro*

**Figure 3:**
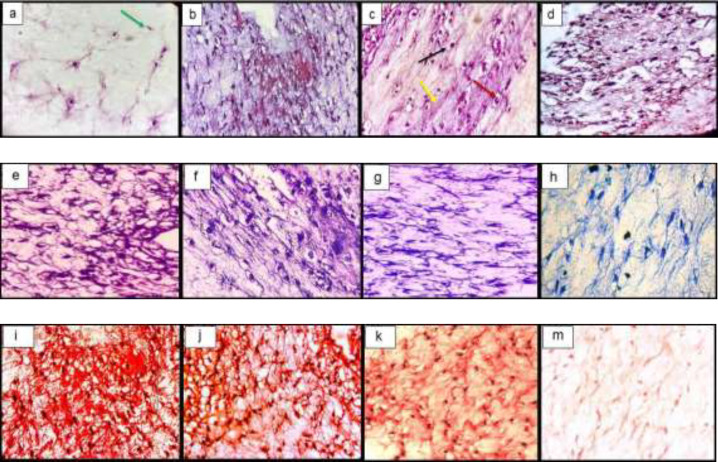
Histological sections of neo-cartilage formed by TGF-β3, KGN and ASU in fibrin scaffold cultures after 14 days as determined by H&E (a: TGF-β3, b: KGN c: ASU and d: Control), Toluidine blue (e: TGF-β3, f: KGN g: ASU and h: Control), Safranin O (i: TGF-β3, j: KGN k: ASU and m: Control) in vitro culture, magnification ×40. Green arrow: Stem cell with an elongated and fusiform nucleus. Red arrow: An isogonic group within Lacuna. Black Arrow: Chondrocytes-like cells inside the lacunae. Yellow arrow: Extracellular matrix

The histological examination of the constructs showed the spreading of chondrocytes shape cells inside the lacuna from the fibrin scaffold and the deposition of the extracellular matrix. Toluidine blue and safranin O stain the GAG so that it is visible. The GAG deposition was higher in TGF-β3, KGN and ASU groups than in the control group (Figure 3).

*Immunohistochemistry (IHC) Assay In-vitro*

**Figure 4:**
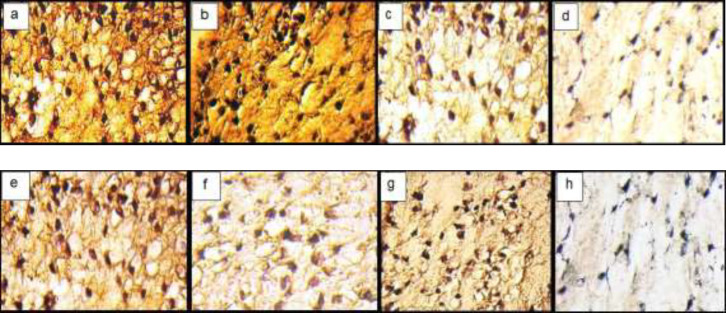
Immunohistochemical sections of neo-cartilage formed by TGF-β3, KGN and ASU in fibrin scaffold. Immunostained with anti-type II collagen antibodies (a: TGF-β3, b: KGN c: ASU and d: Control) and anti-type X collagen antibodies (e: TGF-β3, f: KGN g: ASU and h: Control) in vitro culture, magnification ×40

**Figure 5:**
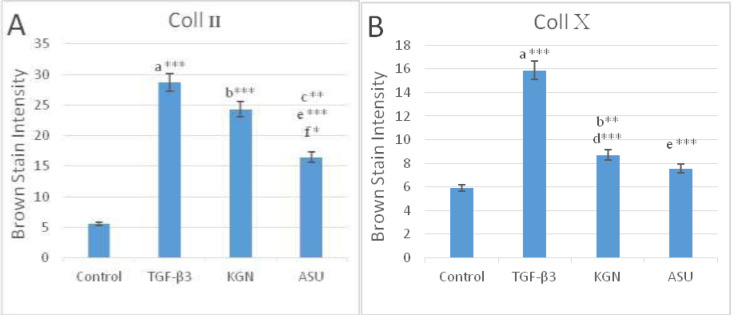
Color intensity demonstrated the deposition of (A) type II and (B) type X collagen protein in control, TGF-β3, KGN and ASU treatment groups. Data are presented as mean ± SD. Error bars represent the standard deviation of the mean. ^*^P < 0.05, ^**^P < 0.01, ^***^P < 0.001

The IHC analyses showed the distribution of Coll II and X in the ECM in fibrin scaffolds both in TGF-β3, KGN and ASU groups. Localization of Coll II in the pre-cellular matrix confirmed that hADSCs chondrogenic differentiation has happened (Figure 4). The average percentage of Coll II positive area was found to be significantly higher in TGF- β3, KGN and ASU groups than in the control group (*p* < 0.05). The Coll X, a cartilage hypertrophic phenotypic marker, was detected in the collagenous matrix of all groups, but in the group that was treated by TGF-β3 deposition, the Coll X was significantly higher than in control, KGN and ASU groups (*P < *0.001) (Figure 5).

*Histological Examination In-vivo*

**Figure 6:**
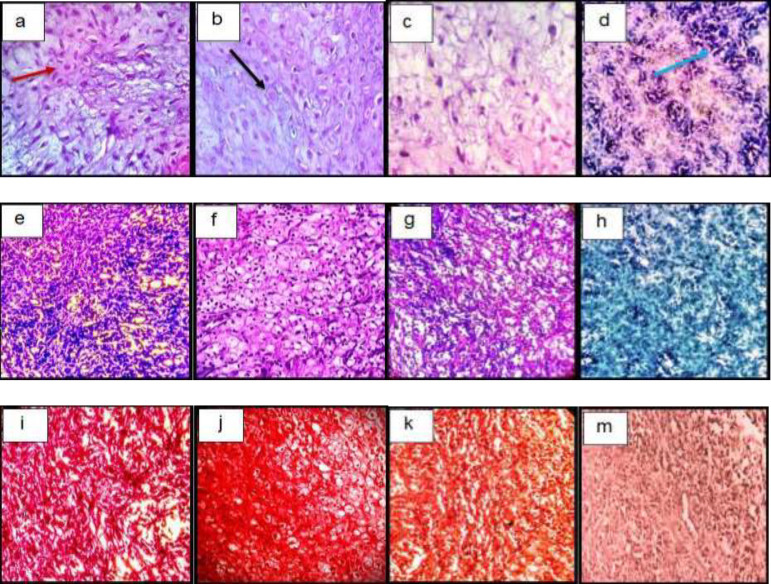
Histological sections of neo-cartilage formed by TGF-β3, KGN and ASU in fibrin scaffold cultures after 14 days as determined by H&E (a: TGF-β3, b: KGN, c: ASU and d: Control), Toluidine blue (e: TGF-β3, f: KGN, g: ASU and h: Control), Safranin O (i: TGF-β3, j: KGN, k: ASU and m: Control) in the rat model, magnification ×40. Blue arrow: Stem cell with an elongated and fusiform nucleus. Red arrow: Extracellular matrix. Black Arrow: Chondrocytes-like cells inside the lacunae

After H&E staining, cells were spherical and similar to chondrocytes, which were single-celled in their lacunae. The basophilic matrix also indicates chondrogenic induction in hADSCs in target groups, and a clear view of clear cartilage was observed in the animal model (Figures 6a-6d).

Semi-quantitative findings of Toluidin Blue staining in target groups indicate the accumulation of acidic glycoproteins in the intercellular matrix in the TGF-β3, KGN, and ASU groups significantly relative to control (*p* < 0.05) (Figures 6f-6h).

Examination of the semi-quantitative findings of his safranin staining showed that the accumulation of GAG in the intercellular matrix in the TGF-β3, KGN, and ASU groups increased significantly compared to control (*p* < 0.05) (Figures 6i-6m).

*Immunohistochemistry Assay In-vivo*

**Figure 7:**
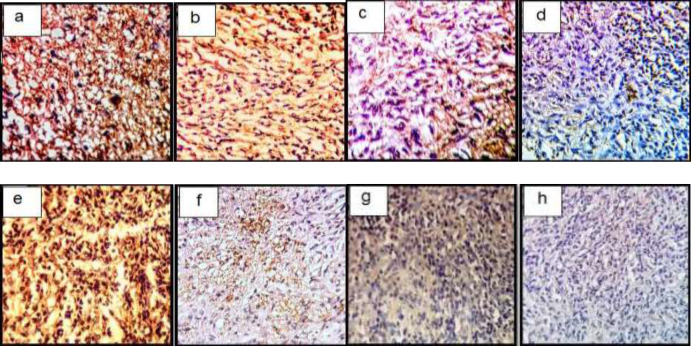
Immunohistochemical sections of neo-cartilage formed by TGF-β3, KGN and ASU in fibrin scaffold cultures after 14 days. Immunostained with anti-type II collagen antibodies (a: TGF-β3, b: KGN, c: ASU and d: Control) and anti-type X collagen antibodies (e: TGF-β3, f: KGN, g: ASU and h: Control) in the rat model, magnification ×40

In a semi-quantitative study of Coll II protein in the intercellular matrix of target groups, a significant increase in the amount of this protein was observed in TGF-β3 and KGN groups compared to control (*p* < 0.05), but in the ASU group compared to control, this increase was not significant (*p* < 0.05) but the presence of Coll II protein in this group was confirmed (Figures 7a-7d and 9).

**Figure 8:**
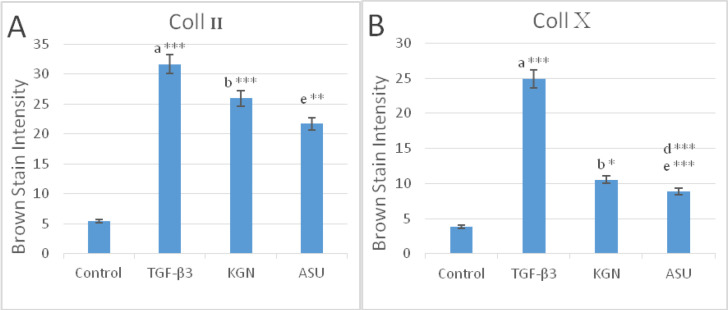
Color intensity demonstrated the deposition of (A) type II and (B) type X collagen protein in control, TGF-β3, KGN, and ASU treatment groups in the rat model. Data are presented as mean ± SD. Error bars represent the standard deviation of the mean. ^*^P < 0.05, ^**^P < 0.01, ^***^P < 0.001

In a semi-quantitative study, the amount of Coll X protein in the intercellular matrix of target groups showed a significant increase in the amount of this protein in the TGF-β3 group compared to other groups (p < 0.05), but in KGN and ASU groups, the difference was significant. There was no control (p < 0.05) (Figures 7e-7h and 8.

## Discussion

In this study, the effects of KGN, ASU and TGF-β3 on the process of chondrogenic differentiation in hADSCs on fibrin scaffold were investigated and compared in two laboratories and animal models. Undoubtedly, articular cartilage defects are one of the most challenging issues in the field of medical science and diseases are considered synovial joint. Due to the intrinsic nature of the cartilage tissue, which is the lack of blood vessels and nerves in the tissue, the repair of this tissue is slow and almost insignificant, apart from a complete lack of healing to activate a subset of inflammatory agents involved in cartilage defects (21, 22). Today, tissue engineering and regenerative medicine aimed to replace lost organs with evolve biotechnologies that combine biomaterials, growth factors, and stem cells. The use of stem cells in the treatment of various diseases is growing.

Among available stem cell sources, ADSCs are an accessible and abundant resource that can be used in cognitive medicine. In 2002, Zuk *et al.* reported that ADSCs differentiate into Cartilage cells under the influence of growth factor β-TGF1 for 14 days was associated with the production of sulfated proteoglycans, which is consistent with our results. Also, after day 14, it was found that the induced cells showed *Coll II *genes, *AGG* and *Coll X*, which is consistent with the current study (23).

Other important components in tissue engineering are cartilage scaffolds that have similar properties to the extracellular matrix. Fibrin scaffolding is one of the natural biomaterials that can be autologous extracted from the patient’s blood (18). This scaffold has been widely used in tissue engineering due to its good biodegradability and biocompatibility, lack of toxicity, enabling cell proliferation and migration, as well as high elasticity (24, 25).

In a 2011 study by Girandon *et al*., to compare proliferation and survival, ADSCs in both fibrin and alginate scaffolds demonstrated that proliferation and survival rates were higher in fibrin scaffolds than in alginate scaffolds. Similarly, apoptosis was lower in this scaffold than in alginate scaffolds (26).

In this study, we used KGN, ASU and TGF-β3 for chondrogenic differentiation. The purpose of these compounds was to find a suitable compound with good chondrogenic differentiation, reasonable price and no harmful side effects, such as causing hypertrophy and reducing stem cell biology.

In the present study, the use of the TGF-β3 growth factor showed that this growth factor could induce chondrogenic differentiation both in-vitro and in an animal model, which confirms the expression of genes involved in chondrogenesis such as *Coll II*, *SOX9* and *AGG*. The results of *in-vitro* expression of these genes and observation of Coll II and X proteins *in-vitro* and in the animal model showed that they were significantly effective against ASU, but this increase was not significant for KGN. Expression of the* Coll X* gene that indicates hypertrophy in differentiated cells in cells treated with chondrogenic media The differentiated TGF-β3 containing cells were significantly increased both in-vitro and in the animal model compared to the other groups, which means that the TGF-β3 growth factor despite its strong influence on chondrocytes stem cells differentiation. It can lead the cells to hypertrophy and possibly apoptosis, which is one of the disadvantages of using this growth factor. We have found in previous research that TGF-β3 increases cell hypertrophy and promotes cartilage to become bony (18). In the present study, the increase in Coll X protein was increased in differentiated cells in the medium containing TGF-β3 in-vitro and in the animal model compared to ASU and KGN groups, which seems to increase the use of TGF-β3 growth factor alone. 

In the present study, ASU was also used as an inducer of chondrogenesis. ASU is a complex combination of many compounds, including fat-soluble vitamins, sterols, triterpenols, and possibly fatty acids (27). Past *in-vitro* and *in-vivo *studies have shown that ASU has a positive effect on osteoarthritis (28) and inhibits cartilage fracture, and stimulates synthesis by inhibiting a number of molecules and pathways involved in osteoarthritis. It becomes collagen and AGG (29). It can also prevent the progression of osteoarthritis by inhibiting inflammatory cytokines such as IL-1, IL-6, IL-8, TNF, and PGE2 by modulating NF-kappaB (30).

Our results showed that after 14 days of ASU *in-vitro*, increased expression of genes involved in chondrogenesis, but this increase was not significant compared to other groups group. Previous research has shown that after 21 days, the expression of genes such as *Coll II* and *AGG* have increased significantly, which is likely due to prolonged use of this factor (7). Decreased expression of *Coll X* can be attributed to the strengths of this inducible factor. This was observed both *in-vitro* and in animal models. Stem cell viability was also higher in the ASU group than in the TGF-β3 and KGN groups, although the molecular process of this event is unclear. It appears that the inhibition of inflammatory factors reported by other researchers prevented cell death and increased cell viability has been fundamental. In 2006, YE Henrotin *et al.* reported that ASU inhibits stem cell differentiation into bone cells, which is exerted through the process of inhibiting inflammatory factors such as matrix metalloproteinase (MMP) and 2-cyclooxygenase and in turn expressing type *Coll I. X* decreases, which is consistent with current research (31).

Nowadays, the small-molecule drug KGN has been suggested as an inducible factor in cartilage tissue engineering, which requires more extensive research. Johnson *et al.* Demonstrated that KGN by binding to the core-binding factor (CBF) -β inhibits the release of this factor from type A and its binding to runt-related transcription factors 1 (RUNX1) and ultimately to the expression of genes involved in chondrogenesis such as *SOX9*, *Coll II*, and *AGG*. It is expressed in rat brain stem cells (10). Scientists’ research suggests that the effect of KGN is on the chondrogenesis process by activating RUNX1. RUNX1 is a protein family of Runt-related transcription factors that RUNX2 and RUNX3 are other members of this family. Their activation initiates DNA replication (32, 33). The results of the present study showed that the use of KGN induces the expression of *Coll II*, *AGG* and *SOX9* genes both *in-vitro* and *in-vivo.* Past research has shown that KGN decreases *Coll X* gene expression by altering the balance from pre-hypertrophy to pre-differentiation and replication (34). In 2014, J.Zhang stated that KGN inhibits the effects of inflammatory factors such as interleukin 1 (IL1) and Tumor necrosis factor β (TNFβ) on chondrocytes in culture and reduces the degenerative process in these cells (16). *SOX9* gene expression was significantly increased in the KGN group compared to the ASU group. However, in the case of *AGG* and *Coll II* and *Coll X* genes expression, this increase was not significant.

 In this study, after 14 days of *in-vitro* chondrogenic differentiation of these cells and the fibrin scaffold in which the cells were differentiated, they were transferred to the skin of male rats, and after 14 days of sampling, histological and immunohistochemical samples were taken the experiment found that the cells had more clear cartilage tissue characteristics than laboratory conditions. In 2008, S. Munirah stated that the amount of glycosaminoglycan in scaffolds containing rabbit chondrocytes was significantly increased after implantation under the skin of mice (35). In 2001, SasanoY *et al.* the expression of *Coll I* in differentiated cells in the animal model was lower than in the laboratory model (36). Comparison of the results of specific staining *in-vitro *and animal models also showed that the differentiation of cells under the skin increased the amount of cartilage matrix constituents such as proteoglycans in the implanted scaffolds and the accumulation rate. Coll X, indicating the onset and occurrence of hypertrophy in differentiated cells, was significantly decreased in some groups, particularly ASU.

**Table 1:** Gene sequence of primers

**Gene**	**Primer sequences (forward and reverse)**
collagen II-F	CTGGTGATGATGGTGAAG
collagen II –R	CCTGGATAACCTCTGTGA
sox-9 –F	TTCAGCAGCCAATAAGTG
sox-9 –R	TTCAGCAGCCAATAAGTG
collagen x –F	AGAATCCATCTGAGAATATGC
collagen x – R	CCTCTTACTGCTATACCTTTAC
Aggrecan-F	GTGGGACTGAAGTTCTTG
Aggrecan-R	GTTGTCATGGTCTGAAGTT
GAPDH-F	AAGCTCATTTCCTGGTATG
GAPDH-R	CTTCCTCTTGTGCTCTTG

## Conclusion

The use of growth factors such as TGF-β3 in cartilage tissue engineering, while expressing specific markers of hyaline cartilage, could cause hypertrophic differentiation of cartilage. Despite the high cost of growth factors, rapid degradation and short half-life, making them widely used, especially in the clinical fields. This study showed that the use of ASU and KGN had positive effects on stem cell proliferation and survival, reduced the chances of chondrocytes hypertrophy, and increased the expression of specific chondrogenic genes in culture and especially in animals.

In conclusion, implantation of differentiated scaffolds and cells prior to transfer to the cartilage lesion under the skin greatly enhances the quality of the cartilage produced and greatly reduces the process of conversion of cartilage to bone. 

## Conflict of interest

There are no conflicts of interest in this research.
